# Supplementary material for: Immune histories and natural infection protection during the omicron era
Source: Commun Med (Lond). 2025 Jul 1;5:262. doi: 10.1038/s43856-025-00974-9 (PMC12215862; doi:10.1038/s43856-025-00974-9)
Supplement: Supplementary file 2 — Supplementary Information [file 43856_2025_974_MOESM2_ESM.pdf]

Supplementary Appendix

Table of Contents

Supplementary methods ... 3

Study population and data sources ... 3

Laboratory methods and variant ascertainment ... 6

COVID-19 severity, criticality, and fatality classification ... 7

Classification of coexisting conditions ... 9

Further details on the matching process ... 10

Supplementary Fig. 1. SARS-CoV-2 infection incidence and variants in Qatar. Daily infection counts and circulating variants from February 5, 2020, to August 12, 2024. ... 12

Supplementary Table 1. Strengthening the Reporting of Observational Studies in Epidemiology (STROBE) checklist for cohort studies. ... 13

Supplementary Fig. 2. Flowchart of the study population selection process in the study comparing the incidence of SARS-CoV-2 reinfection between the omicron double-infection cohort and the omicron single-infection cohort. ... 15

Supplementary Fig. 3. Median dates of immunological events occurring before or after the start of follow-up among individuals who experienced these events in the analyses of A) the omicron single-infection cohort and the omicron double-infection cohort, B) the pre-omicron-omicron double-infection cohort and the omicron double-infection cohort, and C) the omicron single-infection cohort and the pre-omicron-omicron double-infection cohort. Immunological events are not presented in chronological order, as the same type of event, such as vaccination with Dose 4, may occur either before or after the start of follow-up in these individuals. Note Methods section for a detailed description of the study cohorts. ... 16

Supplementary Table 2. Distribution of reinfections by omicron subvariant across the three cohort analyses. ... 17

Supplementary Fig. 4. Cumulative incidence of SARS-CoV-2 reinfection by vaccination status in the studies comparing the incidence of SARS-CoV-2 reinfection between A) the omicron double-infection cohort and the omicron single-infection cohort, B) the omicron double-infection cohort and the pre-omicron-omicron double-infection cohort, and C) the pre-omicron-omicron double-infection cohort and the omicron single-infection cohort. ... 18

Supplementary Fig. 5. Hazard ratios for the incidence of SARS-CoV-2 reinfection by 6-month intervals of follow-up in the studies comparing the incidence of SARS-CoV-2 reinfection between A) the omicron double-infection cohort and the omicron single-infection cohort, B) the omicron double-infection cohort and the pre-omicron-omicron double-infection cohort, and C) the pre-omicron-omicron double-infection cohort and the omicron single-infection cohort. Error bars indicate the corresponding 95% confidence intervals. ... 19

Supplementary Table 3. Subgroup analysis. Hazard ratios for the incidence of SARS-CoV-2 reinfection, stratified by vaccination status, in the comparative analysis of A) the omicron double-infection cohort versus the omicron single-infection cohort, B) the omicron double-infection cohort

versus the pre-omicron-omicron double-infection cohort, and C) the pre-omicron-omicron double-infection cohort versus the omicron single-infection cohort. 20

Supplementary Fig. 6. Flowchart of the study population selection process in the study comparing the incidence of SARS-CoV-2 reinfection between the omicron double-infection cohort and the pre-omicron-omicron double-infection cohort. 22

Supplementary Fig. 7. Flowchart of the study population selection process in the study comparing the incidence of SARS-CoV-2 reinfection between the pre-omicron-omicron double-infection cohort and the omicron single-infection cohort. 23

Supplementary Fig. 8. Sensitivity analysis showing the adjusted cumulative incidence of SARS-CoV-2 reinfection in the studies comparing the incidence of SARS-CoV-2 reinfection between A) the omicron double-infection cohort and the omicron single-infection cohort, B) the omicron double-infection cohort and the pre-omicron-omicron double-infection cohort, and C) the pre-omicron-omicron double-infection cohort and the omicron single-infection cohort. 24

Supplementary Fig. 9. Sensitivity analysis of study outcomes for unvaccinated and vaccinated subgroups using interaction terms. Hazard ratios for the incidence of SARS-CoV-2 reinfection, stratified by vaccination status, in the studies comparing the incidence of SARS-CoV-2 reinfection between A) the omicron double-infection cohort and the omicron single-infection cohort, B) the omicron double-infection cohort and the pre-omicron-omicron double-infection cohort, and C) the pre-omicron-omicron double-infection cohort and the omicron single-infection cohort. Error bars indicate the corresponding 95% confidence intervals. 25

Supplementary References 26

Supplementary methods

Study population and data sources

Qatar's national and universal public healthcare system uses the Cerner Millenium electronic medical record (EMR) system to track all the public healthcare encounters of each individual in the country, including all citizens and residents registered in the national and universal public healthcare system. Registration in the public healthcare system is mandatory for citizens and residents.

The databases analyzed in this study are data-extract downloads from the national EMR that have been implemented on a regular weekly schedule since the onset of pandemic by the Business Intelligence Unit at Hamad Medical Corporation (HMC). HMC is the national public healthcare provider in Qatar. At every download, all severe acute respiratory syndrome coronavirus 2 (SARS-CoV-2) tests, coronavirus disease 2019 (COVID-19) vaccinations, hospitalizations related to COVID-19, and all death records regardless of cause are provided to the authors through .csv files. These databases have been analyzed throughout the pandemic not only for study-related purposes, but also to provide policymakers with summary data and analytics to inform the national response.

Every health encounter in the national EMR is linked to an individual through the HMC Number, which serves as a unique identifier that links all records for this individual at the national level. Databases were merged and analyzed using the HMC Number to link all records pertaining to testing, vaccinations, hospitalizations, and deaths. All deaths in Qatar are recorded by the public healthcare system. COVID-19-related healthcare was provided exclusively in the public healthcare system. COVID-19 vaccination was also provided only through the public healthcare system. These health records were tracked throughout the COVID-19 pandemic using the

national EMR system. This system has been implemented in 2013, before the onset of the pandemic. This pre-established system ensured that we had access to comprehensive health records related to this study for both citizens and residents throughout the entire pandemic, allowing us to follow each person over time.

Demographic details for every HMC Number (individual) such as sex, age, and nationality are collected upon issuing of the universal health card, based on the Qatar Identity Card, which is a mandatory requirement by the Ministry of Interior to every citizen and resident in the country. Data extraction from the Qatar Identity Card to the digital health platform is performed electronically through scanning techniques.

SARS-CoV-2 testing in any facility in Qatar is tracked nationally in one database, the national testing database. This database covers all testing throughout the country, whether in public or private facilities. Every polymerase chain reaction (PCR) test and a proportion of the facility-based rapid antigen tests conducted in Qatar, regardless of location or setting, are classified on the basis of symptoms and the reason for testing, such as the presence of clinical symptoms, contact tracing, participation in surveys or random testing campaigns, individual requests for testing, routine healthcare testing, pre-travel requirements, at the point of entry into the country, or any other relevant reasons for testing.

Before November 1, 2022, SARS-CoV-2 testing in Qatar was performed extensively with about 5% of the population tested every week[1]. Based on the distribution of the reason for testing up to November 1, 2022, most of the tests in Qatar were conducted for routine reasons, such as travel-related purposes, and about 75% of infections were diagnosed not because of presence of symptoms[1,2]. Starting from November 1, 2022, testing for SARS-CoV-2 was substantially

reduced with <1% of the population tested every week[2]. This study factored all SARS-CoV-2-related testing included in the national testing database over the duration of follow-up.

December 19, 2021 marked the onset of the omicron wave in Qatar[1]. The first omicron wave that reached its peak in January of 2022 was massive and strained the testing capacity in the country[1,3-5]. To alleviate the burden on PCR testing, rapid antigen testing was rapidly introduced. The swift change in testing policy precluded incorporating reason for testing for a number of rapid antigen tests. While the reason for testing is documented for all PCR tests, it is not uniformly available for all rapid antigen tests. However, all medically supervised rapid antigen tests were captured by the national integrated digital health platform since January 5, 2022.

Rapid antigen test kits are accessible for purchase at pharmacies in Qatar, but results of home-based testing are neither reported nor documented in the national databases. Since SARS-CoV-2-test outcomes were linked to specific public health measures, restrictions, and privileges, testing policy and guidelines stress facility-based testing as the core testing mechanism in the population. While facility-based testing is provided free of charge or at low subsidized costs, depending on the reason for testing, home-based rapid antigen testing is de-emphasized and not supported as part of national policy.

Qatar launched its COVID-19 vaccination program in December 2020, employing mRNA vaccines and prioritizing individuals based on coexisting conditions and age criteria[2,6]. COVID-19 vaccination was provided free of charge, regardless of citizenship or residency status, and was nationally tracked[2,6].

Qatar has unusually young, diverse demographics, in that only 9% of its residents are ≥50 years of age, and 89% are expatriates from over 150 countries[2,7]. Further descriptions of the study population and these national databases were reported previously[1,2,5,8-11].

Laboratory methods and variant ascertainment

Real-time reverse-transcription polymerase chain reaction testing

Nasopharyngeal and/or oropharyngeal swabs were collected for PCR testing and placed in Universal Transport Medium (UTM). Aliquots of UTM were: 1) extracted on KingFisher Flex (Thermo Fisher Scientific, USA), MGISP-960 (MGI, China), or ExiPrep 96 Lite (Bioneer, South Korea) followed by testing with real-time reverse-transcription PCR (RT-qPCR) using TaqPath COVID-19 Combo Kits (Thermo Fisher Scientific, USA) on an ABI 7500 FAST (Thermo Fisher Scientific, USA); 2) tested directly on the Cepheid GeneXpert system using the Xpert Xpress SARS-CoV-2 (Cepheid, USA); or 3) loaded directly into a Roche cobas 6800 system and assayed with the cobas SARS-CoV-2 Test (Roche, Switzerland). The first assay targets the viral S, N, and ORF1ab gene regions. The second targets the viral N and E-gene regions, and the third targets the ORF1ab and E-gene regions.

All PCR testing was conducted at the Hamad Medical Corporation Central Laboratory or Sidra Medicine Laboratory, following standardized protocols.

Rapid antigen testing

SARS-CoV-2 antigen tests were performed on nasopharyngeal swabs using one of the following lateral flow antigen tests: Panbio COVID-19 Ag Rapid Test Device (Abbott, USA); SARS-CoV-2 Rapid Antigen Test (Roche, Switzerland); Standard Q COVID-19 Antigen Test (SD Biosensor, Korea); or CareStart COVID-19 Antigen Test (Access Bio, USA). All antigen tests were performed at point-of-care according to each manufacturer's instructions, at public or private hospitals and clinics throughout Qatar, with prior authorization and training by the Ministry of Public Health (MOPH). Antigen test results were electronically reported to the MOPH in real

time using the Antigen Test Management System which is integrated with the national COVID-19 database.

Classification of infections by variant type

Surveillance for SARS-CoV-2 variants in Qatar is based on viral genome sequencing and multiplex reverse transcription quantitative PCR (RT-qPCR) variant screening[12] of weekly collected random positive clinical samples[2,13-17], complemented by deep sequencing of wastewater samples[15,18-20]. Further details on the viral genome sequencing and multiplex RT-qPCR variant screening throughout the SARS-CoV-2 waves in Qatar can be found in previous publications[1,2,4,9,13-17,21-26].

COVID-19 severity, criticality, and fatality classification

Classification of COVID-19 case severity (acute-care hospitalizations)[27], criticality (intensive-care-unit hospitalizations)[27], and fatality[28] followed the World Health Organization (WHO) guidelines. Assessments were made by trained medical personnel independent of study investigators and using individual chart reviews, as part of a national protocol applied to every hospitalized COVID-19 patient. Following the national protocol, every individual with a SARS-CoV-2-positive test and a concurrent COVID-19 hospital admission was assessed for infection severity at regular intervals until discharge or death, throughout the pandemic, regardless of the length of hospital stay[29]. Following our earlier studies[29-31], individuals who progress to severe, critical, or fatal COVID-19 between the time of the documented infection and the end of the study are classified based on their worst outcome, starting with death[28], followed by critical disease[27], and then severe disease[27].

Severe COVID-19

Severe COVID-19 disease was defined per WHO classification as a SARS-CoV-2 infected person with "oxygen saturation of <90% on room air, and/or respiratory rate of >30 breaths/minute in adults and children >5 years old (or ≥60 breaths/minute in children <2 months old or ≥50 breaths/minute in children 2-11 months old or ≥40 breaths/minute in children 1–5 years old), and/or signs of severe respiratory distress (accessory muscle use and inability to complete full sentences, and, in children, very severe chest wall indrawing, grunting, central cyanosis, or presence of any other general danger signs)"[27]. Detailed WHO criteria for classifying SARS-CoV-2 infection severity can be found in the WHO technical report[27].

Critical COVID-19

Critical COVID-19 disease was defined per WHO classification as a SARS-CoV-2 infected person with "acute respiratory distress syndrome, sepsis, septic shock, or other conditions that would normally require the provision of life sustaining therapies such as mechanical ventilation (invasive or non-invasive) or vasopressor therapy"[27]. Detailed WHO criteria for classifying SARS-CoV-2 infection criticality can be found in the WHO technical report[27].

Fatal COVID-19

COVID-19 death was defined per WHO classification as "a death resulting from a clinically compatible illness, in a probable or confirmed COVID-19 case, unless there is a clear alternative cause of death that cannot be related to COVID-19 disease (e.g. trauma). There should be no period of complete recovery from COVID-19 between illness and death. A death due to COVID-19 may not be attributed to another disease (e.g. cancer) and should be counted independently of preexisting conditions that are suspected of triggering a severe course of COVID-19". Detailed WHO criteria for classifying COVID-19 death can be found in the WHO technical report[28].

Classification of coexisting conditions

Coexisting conditions were ascertained and classified based on the ICD-10 codes for the conditions, as recorded in the electronic health record encounters of each individual in the national EMR database that includes all citizens and residents registered in the national and universal public healthcare system. The public healthcare system provides healthcare to the entire resident population of Qatar free of charge or at heavily subsidized costs, including prescription drugs. With the mass expansion of this sector in recent years, facilities have been built to cater to specific needs of subpopulations. For example, tens of facilities have been built, including clinics and hospitals, in localities with high density of craft and manual workers[32].

All encounters for each individual were analyzed to determine the coexisting-condition classification for that individual. The national EMR database includes encounters starting from 2013, when this system was launched in Qatar. Any individual who had at least one encounter with a specific coexisting-condition diagnosis since 2013 was classified as having that coexisting condition. Individuals who do not have records of coexisting-condition encounters in the public healthcare system were classified as having no coexisting conditions.

The classification of coexisting conditions spanned the following conditions: 1) Behchet's disease, 2) cancer, 3) cardiovascular diseases, 4) infectious and parasitic diseases, 5) Chron's disease, 6) chronic kidney disease (CKD), 7) chronic liver disease (CLD), 8) chronic lung disease, 9) congenital malformations, deformations and chromosomal abnormalities, 10) diseases of the blood and blood-forming organs and certain disorders involving the immune mechanism, 11) diseases of the ear and mastoid process, 12) deep vein thrombosis (DVT), 13) dermatitis, 14) diabetes mellitus, 15) diseases of the circulatory system, 16) diseases of the digestive system, 17) diseases of the eye and adnex, 18) diseases of the genitourinary system, 19) diseases of the

musculoskeletal system and connective tissue, 20) diseases of the nervous system, 21) diseases of the respiratory system, 22) diseases of the skin and subcutaneous tissue, 23) endocrine, nutritional and metabolic diseases, 24) gingivitis, 25) hypertension, 26) injury, poisoning and certain other consequences of external causes, 27) mental and behavioral disorders, 28) neoplasms, 29 periodontitis, 30) pregnancy, childbirth and the puerperium, 31) pulmonary tuberculosis, 32) rheumatoid arthritis, 33) Sjogren's syndrome, 34) stroke or neural conditions, 35) symptoms, signs and abnormal clinical and laboratory findings, not elsewhere classified, 36) systemic lupus erythematosus, 37) systemic sclerosis, 38) organ transplant, and 39) other unspecified factors influencing health status and contact with health services.

Further details on the matching process

Matching by calendar time was implemented to ensure that matched pairs were present in Qatar during the same period. In the first study, individuals in the omicron double-infection cohort whose reinfection occurred in a specific calendar week were matched to individuals in the omicron single-infection cohort whose primary infection occurred during that same week. In this study, individuals in the omicron double-infection cohort may have contributed follow-up time as part of the matched omicron single-infection cohort, after their primary omicron infection and before their omicron reinfection, and subsequently, as part of the omicron double-infection cohort after reinfection. This means that some individuals could have been matched more than once due to changes in their eligibility for inclusion in different cohorts (a cross-over design). However, no individual contributed to the two cohorts *simultaneously* under the same inclusion criteria.

In the second study, individuals in the omicron double-infection cohort whose reinfection occurred in a specific calendar week were matched to individuals in the pre-omicron-omicron

double-infection cohort whose reinfection occurred during that same week. Due to differing primary infection histories, there was no possibility of overlap between individuals in these cohorts.

In the third study, individuals in the pre-omicron-omicron double-infection cohort whose reinfection occurred in a specific calendar week were matched to individuals in the omicron single-infection cohort whose primary infection occurred during that same week. Due to differing primary infection histories, there was no possibility of overlap between individuals in these cohorts.

In all analyses, individuals who were tested after death, had an unascertained or discrepant death date, or died before the start of follow-up were excluded.

The matching approach for this study aimed to balance observed confounders that could potentially affect the risk of infection across the exposure groups[2,32-35]. The matching factors were selected based on findings of earlier COVID-19 epidemiologic studies on Qatar's population[2,10,36-40].

The matching algorithm was implemented using *ccmatch* command in Stata 18.0 supplemented with conditions to retain only controls that fulfilled the eligibility criteria, and was iterated using loops with as many replications as needed until exhaustion (i.e., no more matched pairs could be identified).

Supplementary Fig. 1. SARS-CoV-2 infection incidence and variants in Qatar. Daily infection counts and circulating variants from February 5, 2020, to August 12, 2024.

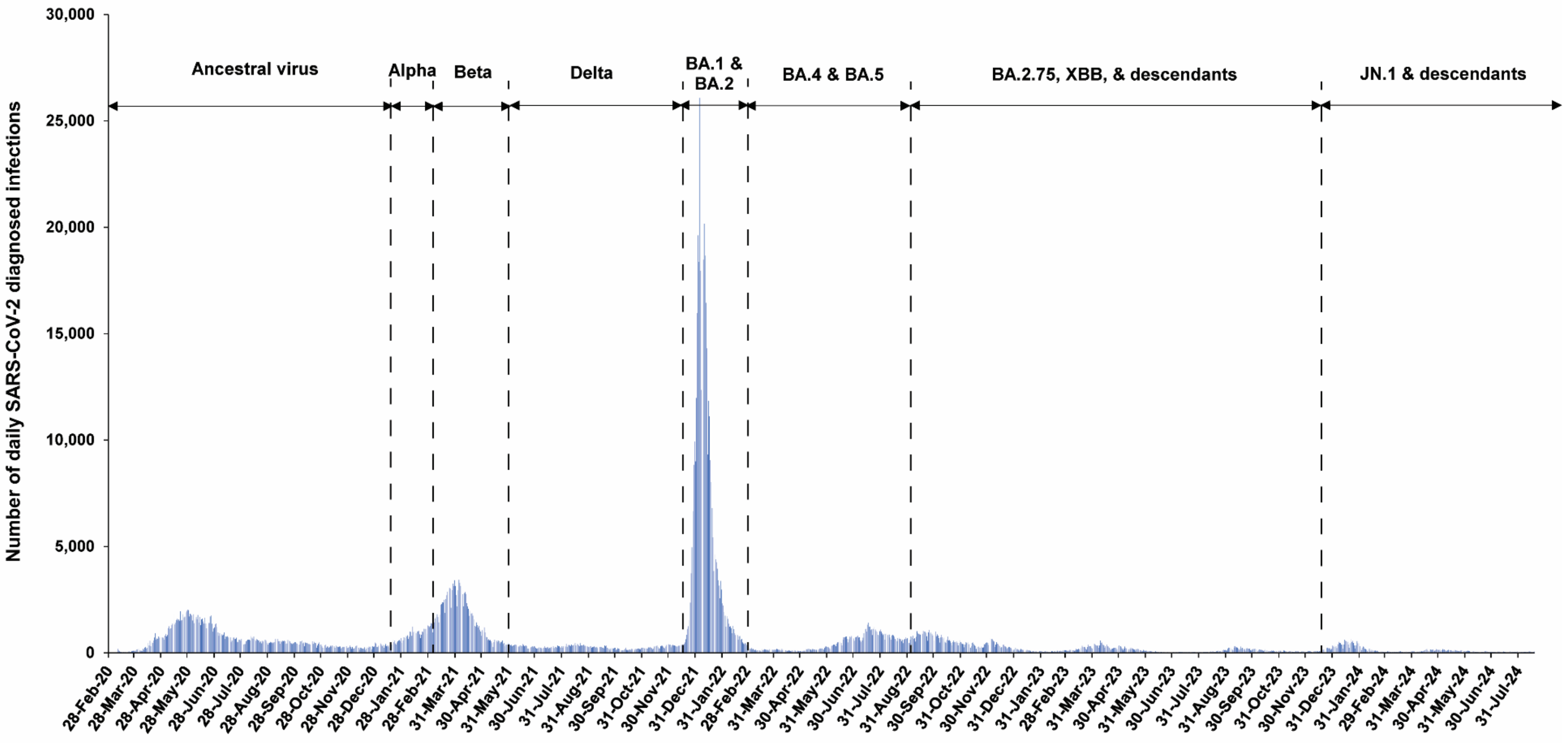

Supplementary Table 1. Strengthening the Reporting of Observational Studies in Epidemiology (STROBE) checklist for cohort studies.

	Item No	Recommendation	Main Text page
**Title and abstract**	1	(*a*) Indicate the study's design with a commonly used term in the title or the abstract	Abstract
		(*b*) Provide in the abstract an informative and balanced summary of what was done and what was found	Abstract
**Introduction**			
Background/rationale	2	Explain the scientific background and rationale for the investigation being reported	Introduction
Objectives	3	State specific objectives, including any prespecified hypotheses	Introduction
**Methods**			
Study design	4	Present key elements of study design early in the paper	Methods ('Study population, data sources, and vaccination' & 'Study design') & Fig. 1
Setting	5	Describe the setting, locations, and relevant dates, including periods of recruitment, exposure, follow-up, and data collection	Methods ('Study population, data sources, and vaccination', 'Study design', & 'Cohorts' follow-up'), Supplementary methods, & Supplementary Figs. 2, 6 & 7
Participants	6	(*a*) Give the eligibility criteria, and the sources and methods of selection of participants. Describe methods of follow-up<br>(*b*) For matched studies, give matching criteria and number of exposed and unexposed	Methods ('Study design', 'Cohorts' matching' & 'Cohorts' follow-up'), & Supplementary Figs. 2, 6 & 7, & Supplementary methods
Variables	7	Clearly define all outcomes, exposures, predictors, potential confounders, and effect modifiers. Give diagnostic criteria, if applicable	Methods ('Study design', 'Cohorts' matching' & 'Cohorts' follow-up', & 'Statistical analysis'), Supplementary Data 1, & Supplementary methods
Data sources/ measurement	8*	For each variable of interest, give sources of data and details of methods of assessment (measurement). Describe comparability of assessment methods if there is more than one group	Methods ('Study population, data sources, and vaccination', 'Study design', & 'Statistical analysis'), Supplementary Data 1, & Supplementary methods
Bias	9	Describe any efforts to address potential sources of bias	Methods ('Cohorts' matching', 'Cohorts' follow-up', & 'Statistical analysis')
Study size	10	Explain how the study size was arrived at	Supplementary Figs. 2, 6 & 7
Quantitative variables	11	Explain how quantitative variables were handled in the analyses. If applicable, describe which groupings were chosen and why	Methods ('Cohorts' matching' & 'Statistical analysis') & Supplementary Data 1
Statistical methods	12	(*a*) Describe all statistical methods, including those used to control for confounding	Methods ('Statistical analysis')
		(*b*) Describe any methods used to examine subgroups and interactions	Methods ('Statistical analysis')
		(*c*) Explain how missing data were addressed	Not applicable, see Methods ('Study population, data sources, and vaccination') & Supplementary methods
		(*d*) If applicable, explain how loss to follow-up was addressed	Not applicable, see Methods ('Study population, data sources, and vaccination') & Supplementary methods
		(*e*) Describe any sensitivity analyses	Not applicable
**Results**			
Participants	13*	(a) Report numbers of individuals at each stage of study—eg numbers potentially eligible, examined for eligibility, confirmed eligible, included in the study, completing follow-up, and analysed<br>(b) Give reasons for non-participation at each stage<br>(c) Consider use of a flow diagram	Supplementary Figs. 2, 6 & 7
Descriptive data	14	(a) Give characteristics of study participants (eg demographic, clinical, social) and information on exposures and potential confounders	Table 1
		(b) Indicate number of participants with missing data for each variable of interest	Not applicable, see Methods ('Study population, data sources,

			and vaccination') & Supplementary methods
		(c) Summarise follow-up time (eg, average and total amount)	Fig. 2 and Table 1
Outcome data	15	Report numbers of outcome events or summary measures over time	Results ('Omicron double-infection cohort and omicron single-infection cohort', paragraph 3, 'Omicron double-infection cohort and pre-omicron-omicron double-infection cohort', paragraph 3, & 'Pre-omicron-omicron double-infection cohort and omicron single-infection cohort', paragraph 3), Table 1, & Supplementary Figs. 2, 6 & 7
Main results	16	(a) Give unadjusted estimates and, if applicable, confounder-adjusted estimates and their precision (eg, 95% confidence interval). Make clear which confounders were adjusted for and why they were included	Results ('Omicron double-infection cohort and omicron single-infection cohort', paragraph 4, 'Omicron double-infection cohort and pre-omicron-omicron double-infection cohort', paragraph 4, & 'Pre-omicron-omicron double-infection cohort and omicron single-infection cohort', paragraph 4), & Table 1
		(b) Report category boundaries when continuous variables were categorized	Supplementary Data 1
		(c) If relevant, consider translating estimates of relative risk into absolute risk for a meaningful time period	Not applicable
Other analyses	17	Report other analyses done—eg analyses of subgroups and interactions, and sensitivity analyses	Results ('Omicron double-infection cohort and omicron single-infection cohort', paragraph 4, 'Omicron double-infection cohort and pre-omicron-omicron double-infection cohort', paragraph 4, & 'Pre-omicron-omicron double-infection cohort and omicron single-infection cohort', paragraph 4), Fig. 3, Supplementary Tables 2 & 3, & Supplementary Figs. 3-5 & 8-9
Discussion			
Key results	18	Summarise key results with reference to study objectives	Discussion, paragraphs 1-5
Limitations	19	Discuss limitations of the study, taking into account sources of potential bias or imprecision. Discuss both direction and magnitude of any potential bias	Discussion, paragraphs 6-12
Interpretation	20	Give a cautious overall interpretation of results considering objectives, limitations, multiplicity of analyses, results from similar studies, and other relevant evidence	Discussion, paragraph 14
Generalisability	21	Discuss the generalisability (external validity) of the study results	Discussion, paragraph 8
Other information			
Funding	22	Give the source of funding and the role of the funders for the present study and, if applicable, for the original study on which the present article is based	Acknowledgments

Supplementary Fig. 2. Flowchart of the study population selection process in the study comparing the incidence of SARS-CoV-2 reinfection between the omicron double-infection cohort and the omicron single-infection cohort.

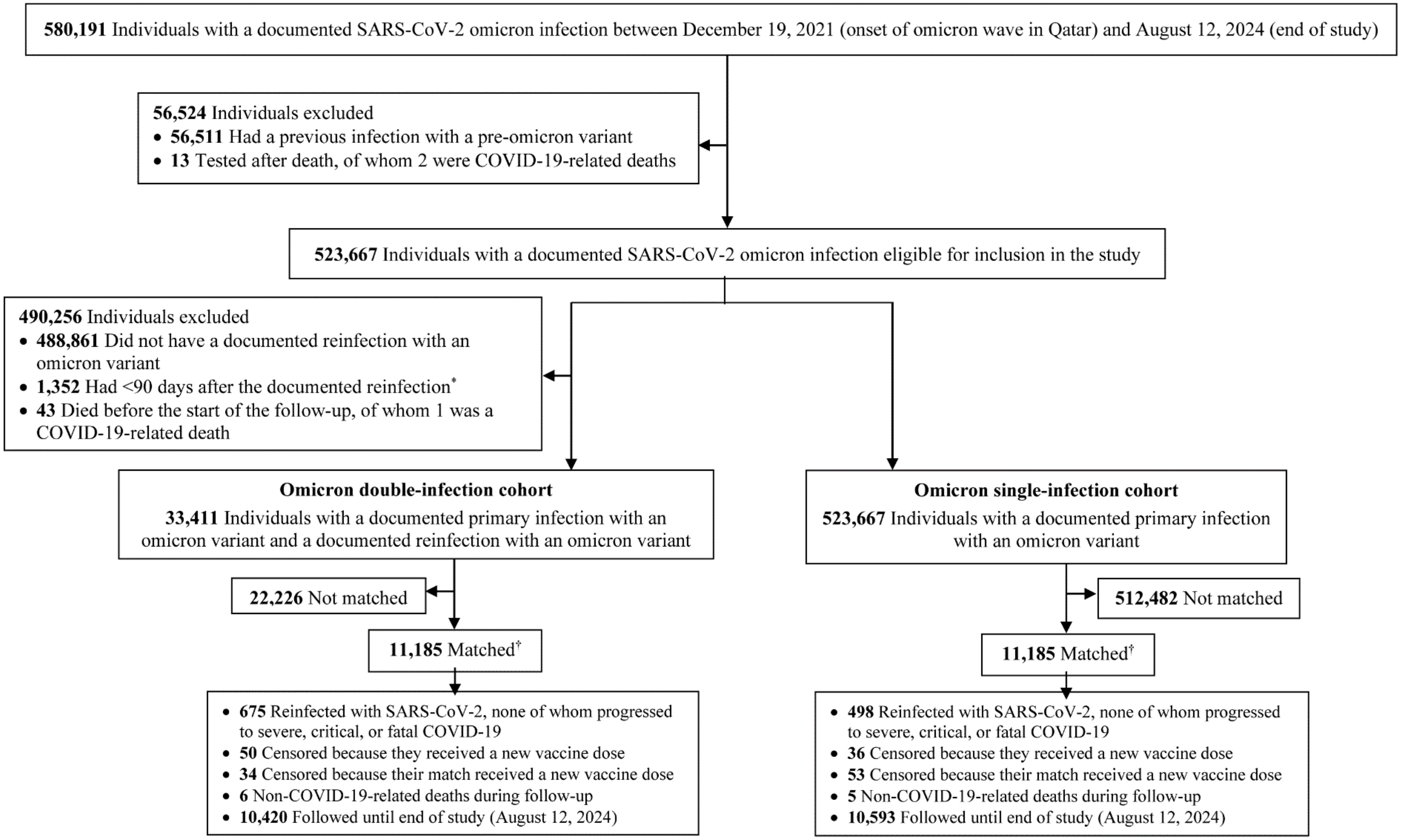

Abbreviations: COVID-19, coronavirus diseases 2019, PCR, polymerase chain reaction, SARS-CoV-2 severe acute respiratory syndrome coronavirus 2.

*A reinfection was defined as a SARS-CoV-2-positive test that is ≥90 days after the previous SARS-CoV-2-positive test.

†Persons in the omicron double-infection cohort were matched exactly one-to-one to persons in the omicron single-infection cohort by sex, 10-year age group, nationality, number of coexisting conditions, number of vaccine doses, vaccine type, in addition to testing method (PCR versus rapid-antigen testing), reason for testing, and calendar week of the SARS-CoV-2-positive test defining the reinfection with omicron for the omicron double-infection cohort and defining the primary infection with omicron for the omicron single-infection cohort. Each matched pair was followed from 90 days after the date of the reinfection for the individual in the omicron double-infection cohort.

Supplementary Fig. 3. Median dates of immunological events occurring before or after the start of follow-up among individuals who experienced these events in the analyses of A) the omicron single-infection cohort and the omicron double-infection cohort, B) the pre-omicron-omicron double-infection cohort and the omicron double-infection cohort, and C) the omicron single-infection cohort and the pre-omicron-omicron double-infection cohort. Immunological events are not presented in chronological order, as the same type of event, such as vaccination with Dose 4, may occur either before or after the start of follow-up in these individuals. Note Methods section for a detailed description of the study cohorts.

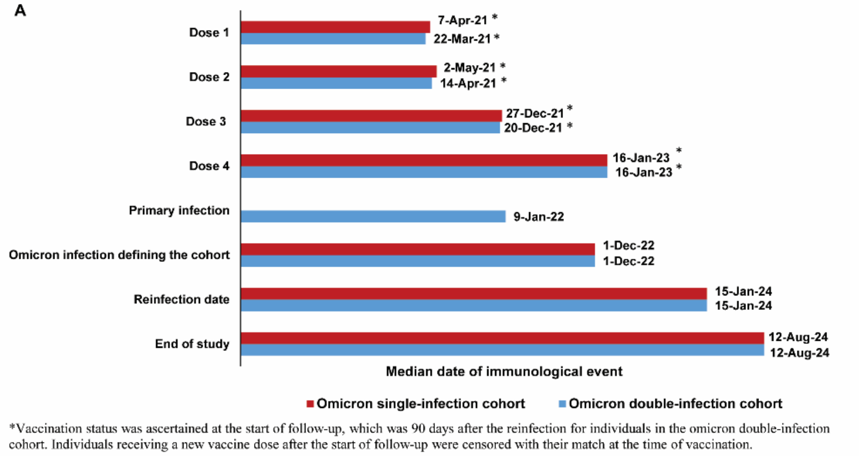

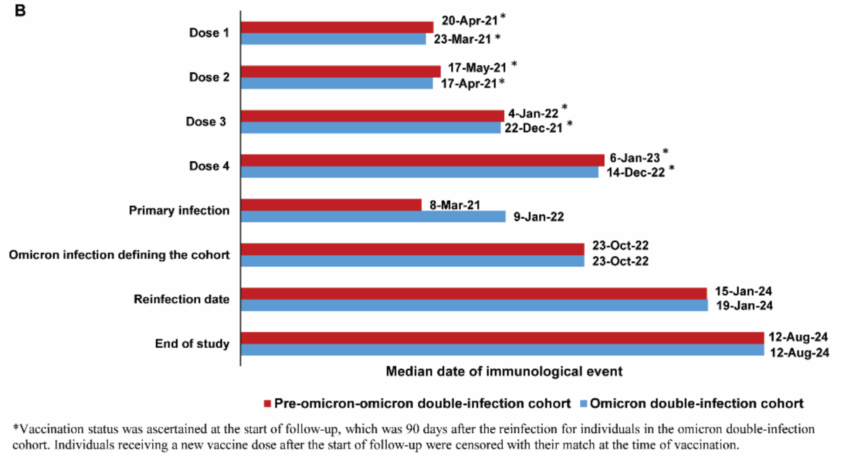

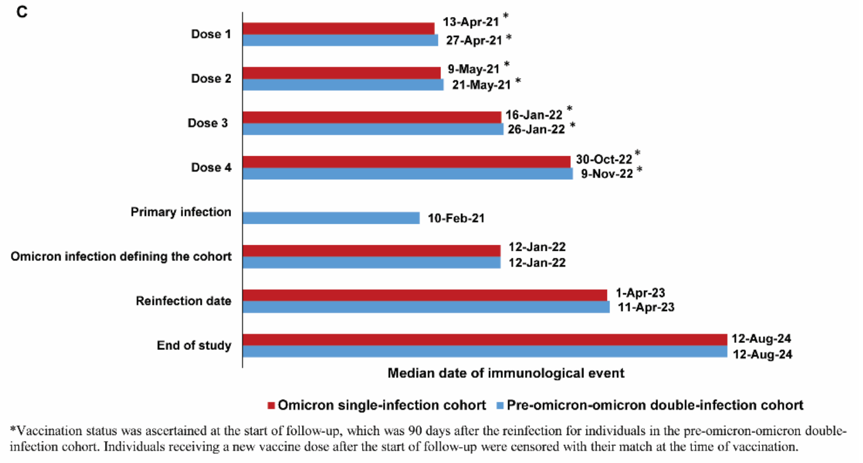

Supplementary Table 2. Distribution of reinfections by omicron subvariant across the three cohort analyses.

SARS-CoV-2 variant	Omicron double-infection cohort versus omicron single-infection cohort[*]			Omicron double-infection cohort versus pre-omicron-omicron double-infection cohort[†]			Pre-omicron-omicron double-infection cohort versus omicron single-infection cohort[‡]		
	Omicron double-infection cohort	Omicron single-infection cohort	SMD[§]	Omicron double-infection cohort	Pre-omicron-omicron double-infection cohort	SMD[§]	Pre-omicron-omicron double-infection cohort	Omicron single-infection cohort	SMD[§]
	N=675	N=498		N=268	N=185		N=2,784	N=2,726	
BA.1 & BA.2	--	--	0.09	--	--	0.12	--	--	0.09
BA.4 & BA.5	--	--		--	--		341 (12.2)	401 (14.7)	
BA.2.75, XBB & descendants	16 (2.4)	6 (1.2)		5 (1.9)	1 (0.5)		548 (19.7)	587 (21.5)	
JN.1 & descendants	659 (97.6)	492 (98.8)		263 (98.1)	184 (99.5)		1,895 (68.1)	1,738 (63.8)	

Abbreviations: SARS-CoV-2, severe acute respiratory syndrome coronavirus 2, PCR, polymerase chain reaction, SMD, standardized mean difference.

[*]Persons in the omicron double-infection cohort were matched exactly one-to-one to persons in the omicron single-infection cohort by sex, 10-year age group, nationality, number of coexisting conditions, number of vaccine doses, vaccine type, in addition to testing method (PCR versus rapid-antigen testing), reason for testing, and calendar week of the SARS-CoV-2-positive test defining the reinfection with omicron for the omicron double-infection cohort and defining the primary infection with omicron for the omicron single-infection cohort. Each matched pair was followed from 90 days after the date of the reinfection for the individual in the omicron double-infection cohort.

[†]Persons in the omicron double-infection cohort were matched exactly one-to-one to persons in the pre-omicron-omicron double-infection cohort by sex, 10-year age group, nationality, number of coexisting conditions, number of vaccine doses, vaccine type, in addition to testing method (PCR versus rapid-antigen testing), reason for testing, and calendar week of the SARS-CoV-2-positive test defining the reinfection with omicron for the omicron double-infection cohort and the pre-omicron-omicron double-infection cohort. Each matched pair was followed from 90 days after the date of the reinfection for the individual in the omicron double-infection cohort.

[‡]Persons in the pre-omicron-omicron double-infection cohort were matched exactly one-to-one to persons in the omicron single-infection cohort by sex, 10-year age group, nationality, number of coexisting conditions, number of vaccine doses, vaccine type, in addition to testing method (PCR versus rapid-antigen testing), reason for testing, and calendar week of the SARS-CoV-2-positive test defining the reinfection with omicron for the pre-omicron-omicron double-infection cohort and defining the primary infection with omicron for the omicron single-infection cohort. Each matched pair was followed from 90 days after the date of the reinfection for the individual in the pre-omicron-omicron double-infection cohort.

[§]SMD is the difference in the mean of a covariate between groups divided by the pooled standard deviation. An SMD ≤0.1 indicates adequate matching.

Supplementary Fig. 4. Cumulative incidence of SARS-CoV-2 reinfection by vaccination status in the studies comparing the incidence of SARS-CoV-2 reinfection between A) the omicron double-infection cohort and the omicron single-infection cohort, B) the omicron double-infection cohort and the pre-omicron-omicron double-infection cohort, and C) the pre-omicron-omicron double-infection cohort and the omicron single-infection cohort.

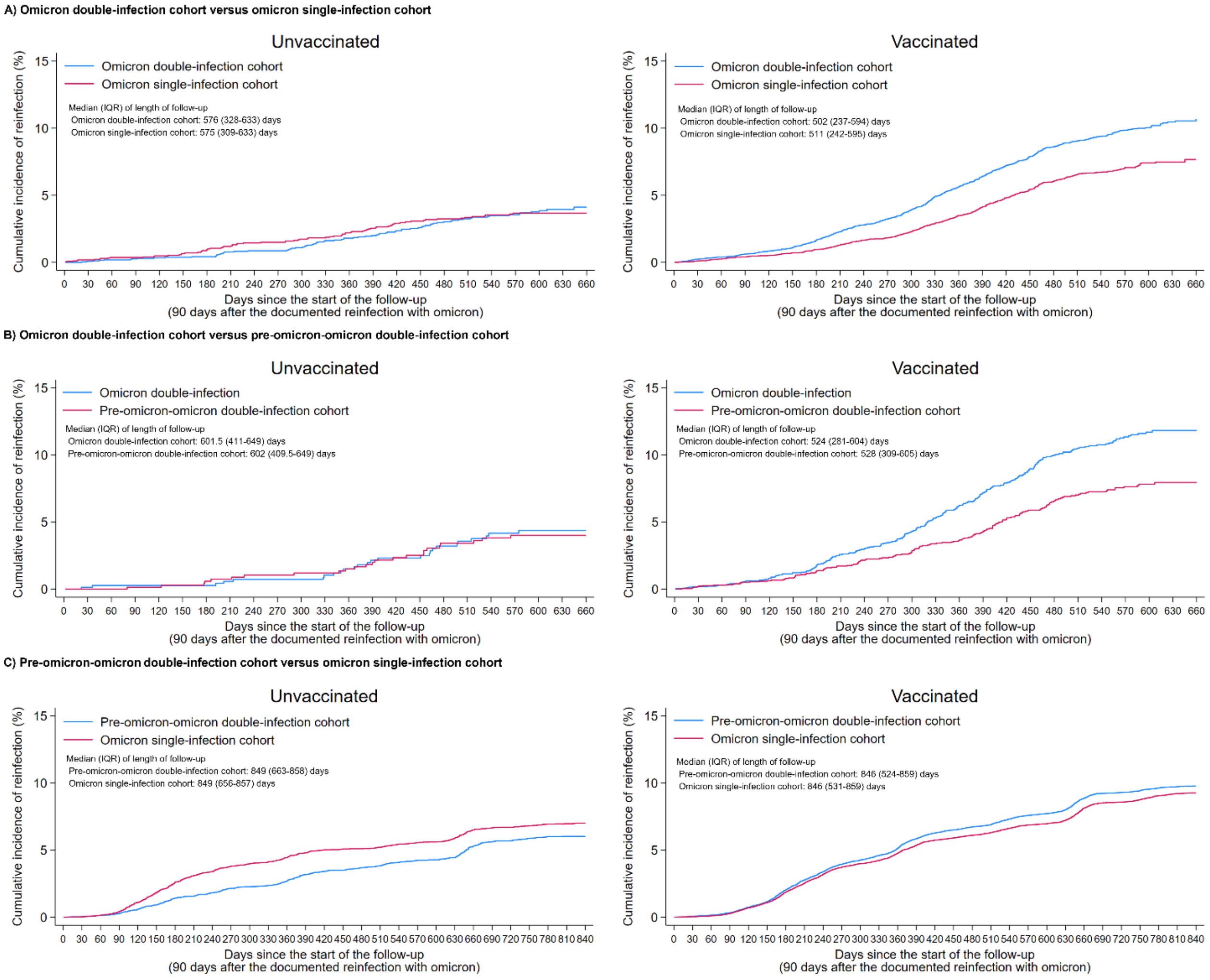

Abbreviations: IQR, interquartile range.

Supplementary Fig. 5. Hazard ratios for the incidence of SARS-CoV-2 reinfection by 6-month intervals of follow-up in the studies comparing the incidence of SARS-CoV-2 reinfection between A) the omicron double-infection cohort and the omicron single-infection cohort, B) the omicron double-infection cohort and the pre-omicron-omicron double-infection cohort, and C) the pre-omicron-omicron double-infection cohort and the omicron single-infection cohort. Error bars indicate the corresponding 95% confidence intervals.

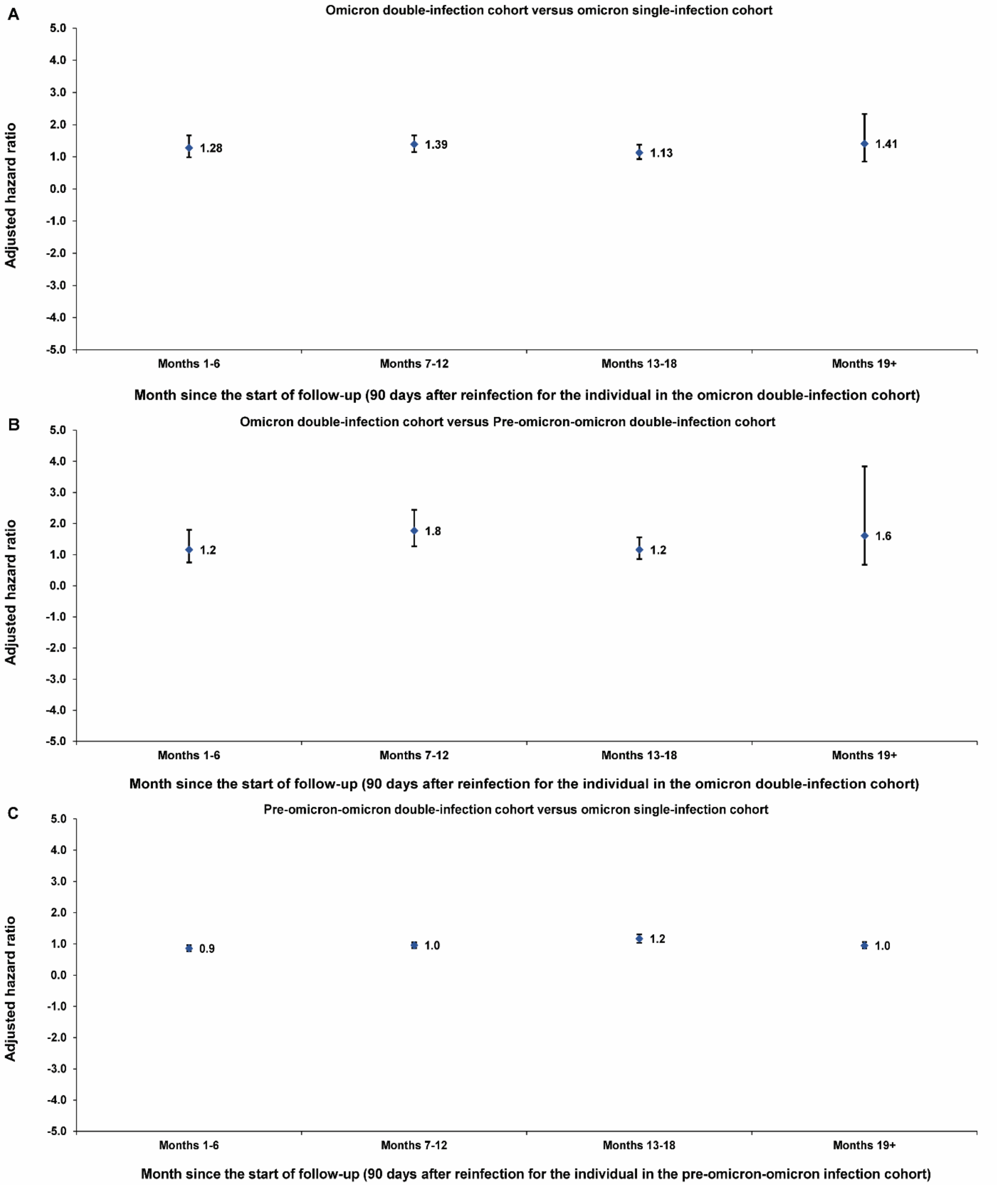

Supplementary Table 3. Subgroup analysis. Hazard ratios for the incidence of SARS-CoV-2 reinfection, stratified by vaccination status, in the comparative analysis of A) the omicron double-infection cohort versus the omicron single-infection cohort, B) the omicron double-infection cohort versus the pre-omicron-omicron double-infection cohort, and C) the pre-omicron-omicron double-infection cohort versus the omicron single-infection cohort.

A) Omicron double-infection cohort versus omicron single-infection cohort

Epidemiological measure	Omicron double-infection cohort[*]	Omicron single-infection cohort[*]
**Unvaccinated**		
Unadjusted hazard ratio for SARS-CoV-2 reinfection (95% CI)	1.00 (0.73 to 1.36)	
Adjusted hazard ratio for SARS-CoV-2 reinfection (95% CI)[†]	0.93 (0.68 to 1.28)	
**Vaccinated**		
Unadjusted hazard ratio for SARS-CoV-2 reinfection (95% CI)	1.44 (1.27 to 1.63)	
Adjusted hazard ratio for SARS-CoV-2 reinfection (95% CI)[†]	1.34 (1.18 to 1.52)	
**Primary-series**		
Unadjusted hazard ratio for SARS-CoV-2 reinfection (95% CI)	1.39 (1.18 to 1.65)	
Adjusted hazard ratio for SARS-CoV-2 reinfection (95% CI)[†]	1.37 (1.16 to 1.63)	
**Three or more doses**		
Unadjusted hazard ratio for SARS-CoV-2 reinfection (95% CI)	1.51 (1.25 to 1.82)	
Adjusted hazard ratio for SARS-CoV-2 reinfection (95% CI)[†]	1.31 (1.08 to 1.58)	

B) Omicron double-infection cohort versus pre-omicron-omicron double-infection cohort

Epidemiological measure	Omicron double-infection cohort[‡]	Pre-omicron-omicron double-infection cohort[‡]
**Unvaccinated**		
Unadjusted hazard ratio for SARS-CoV-2 reinfection (95% CI)	1.08 (0.62 to 1.88)	
Adjusted hazard ratio for SARS-CoV-2 reinfection (95% CI)[§]	1.12 (0.63 to 1.97)	
**Vaccinated**		
Unadjusted hazard ratio for SARS-CoV-2 reinfection (95% CI)	1.53 (1.25 to 1.87)	
Adjusted hazard ratio for SARS-CoV-2 reinfection (95% CI)[§]	1.42 (1.16 to 1.74)	
**Primary-series**		
Unadjusted hazard ratio for SARS-CoV-2 reinfection (95% CI)	1.46 (1.13 to 1.88)	
Adjusted hazard ratio for SARS-CoV-2 reinfection (95% CI)[§]	1.37 (1.06 to 1.77)	
**Three or more doses**		
Unadjusted hazard ratio for SARS-CoV-2 reinfection (95% CI)	1.69 (1.22 to 2.33)	
Adjusted hazard ratio for SARS-CoV-2 reinfection (95% CI)[§]	1.39 (0.99 to 1.94)	

C) Pre-omicron-omicron double-infection cohort versus omicron single-infection cohort

Epidemiological measure	Pre-omicron-omicron double-infection cohort[¦]	Omicron single-infection cohort[¦]
**Unvaccinated**		
Unadjusted hazard ratio for SARS-CoV-2 reinfection (95% CI)	0.83 (0.74 to 0.94)	
Adjusted hazard ratio for SARS-CoV-2 reinfection (95% CI)[¶]	0.75 (0.66 to 0.85)	
**Vaccinated**		
Unadjusted hazard ratio for SARS-CoV-2 reinfection (95% CI)	1.07 (1.01 to 1.13)	
Adjusted hazard ratio for SARS-CoV-2 reinfection (95% CI)[¶]	1.03 (0.97 to 1.09)	
**Primary-series**		
Unadjusted hazard ratio for SARS-CoV-2 reinfection (95% CI)	1.06 (0.99 to 1.14)	
Adjusted hazard ratio for SARS-CoV-2 reinfection (95% CI)[¶]	1.03 (0.96 to 1.10)	
**Three or more doses**		

Unadjusted hazard ratio for SARS-CoV-2 reinfection (95% CI)	1.09 (0.98 to 1.22)
Adjusted hazard ratio for SARS-CoV-2 reinfection (95% CI)[¶]	1.05 (0.94 to 1.17)

Abbreviations: CI, confidence interval, PCR, polymerase chain reaction, SARS-CoV-2 severe acute respiratory syndrome coronavirus 2.

[*]Persons in the omicron double-infection cohort were matched exactly one-to-one to persons in the omicron single-infection cohort by sex, 10-year age group, nationality, number of coexisting conditions, number of vaccine doses, vaccine type, in addition to testing method (PCR versus rapid-antigen testing), reason for testing, and calendar week of the SARS-CoV-2-positive test defining the reinfection with omicron for the omicron double-infection cohort and defining the primary infection with omicron for the omicron single-infection cohort. Each matched pair was followed from 90 days after the date of the reinfection for the individual in the omicron double-infection cohort.

[†]Adjusted for sex, 10-year age group, nationality, number of coexisting conditions, testing method, reason for testing, and calendar week of the SARS-CoV-2-positive test defining the reinfection with omicron for the omicron double-infection cohort and defining the primary infection with omicron for the omicron single-infection cohort, testing rate, and where applicable (vaccinated stratum), for number of vaccine doses and vaccine type.

[‡]Persons in the omicron double-infection cohort were matched exactly one-to-one to persons in the pre-omicron-omicron double-infection cohort by sex, 10-year age group, nationality, number of coexisting conditions, number of vaccine doses, vaccine type, in addition to testing method (PCR versus rapid-antigen testing), reason for testing, and calendar week of the SARS-CoV-2-positive test defining the reinfection with omicron for the omicron double-infection cohort and the pre-omicron-omicron double-infection cohort. Each matched pair was followed from 90 days after the date of the reinfection for the individual in the omicron double-infection cohort.

[§]Adjusted for sex, 10-year age group, nationality, number of coexisting conditions, testing method, reason for testing, and calendar week of the SARS-CoV-2-positive test defining the reinfection with omicron for the omicron double-infection cohort and for the pre-omicron-omicron double-infection cohort, testing rate, and where applicable (vaccinated stratum), for number of vaccine doses and vaccine type.

[‖]Persons in the pre-omicron-omicron double-infection cohort were matched exactly one-to-one to persons in the omicron single-infection cohort by sex, 10-year age group, nationality, number of coexisting conditions, number of vaccine doses, vaccine type, in addition to testing method (PCR versus rapid-antigen testing), reason for testing, and calendar week of the SARS-CoV-2-positive test defining the reinfection with omicron for the pre-omicron-omicron double-infection cohort and defining the primary infection with omicron for the omicron single-infection cohort. Each matched pair was followed from 90 days after the date of the reinfection for the individual in the pre-omicron-omicron double-infection cohort.

[¶]Adjusted for sex, 10-year age group, nationality, number of coexisting conditions, testing method, reason for testing, and calendar week of the SARS-CoV-2-positive test defining the reinfection with omicron for the pre-omicron-omicron double-infection cohort and defining the primary infection with omicron for the omicron single-infection cohort, testing rate, and where applicable (vaccinated stratum), for number of vaccine doses and vaccine type.

Supplementary Fig. 6. Flowchart of the study population selection process in the study comparing the incidence of SARS-CoV-2 reinfection between the omicron double-infection cohort and the pre-omicron-omicron double-infection cohort.

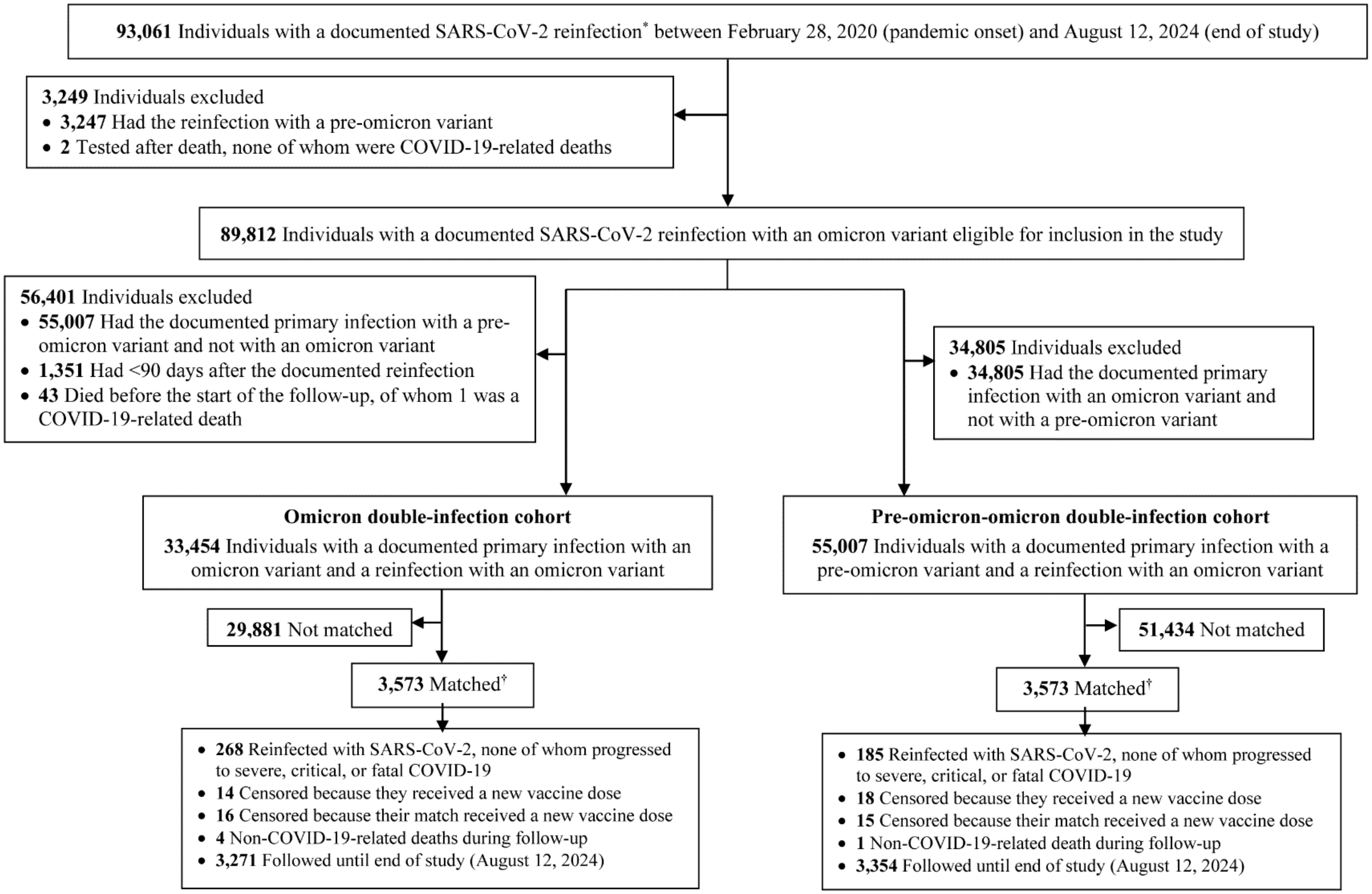

Abbreviations: COVID-19, coronavirus diseases 2019, PCR, polymerase chain reaction, SARS-CoV-2 severe acute respiratory syndrome coronavirus 2.

*A reinfection was defined as a positive SARS-CoV-2 test that is ≥90 days after the first positive test.

†Persons in the omicron double-infection cohort were matched exactly one-to-one to persons in the pre-omicron-omicron double-infection cohort by sex, 10-year age group, nationality, number of coexisting conditions, number of vaccine doses, vaccine type, in addition to testing method (PCR versus rapid-antigen testing), reason for testing, and calendar week of the SARS-CoV-2-positive test defining the reinfection with omicron for the omicron double-infection cohort and the pre-omicron-omicron double-infection cohort. Each matched pair was followed from 90 days after the date of the reinfection for the individual in the omicron double-infection cohort.

Supplementary Fig. 7. Flowchart of the study population selection process in the study comparing the incidence of SARS-CoV-2 reinfection between the pre-omicron-omicron double-infection cohort and the omicron single-infection cohort.

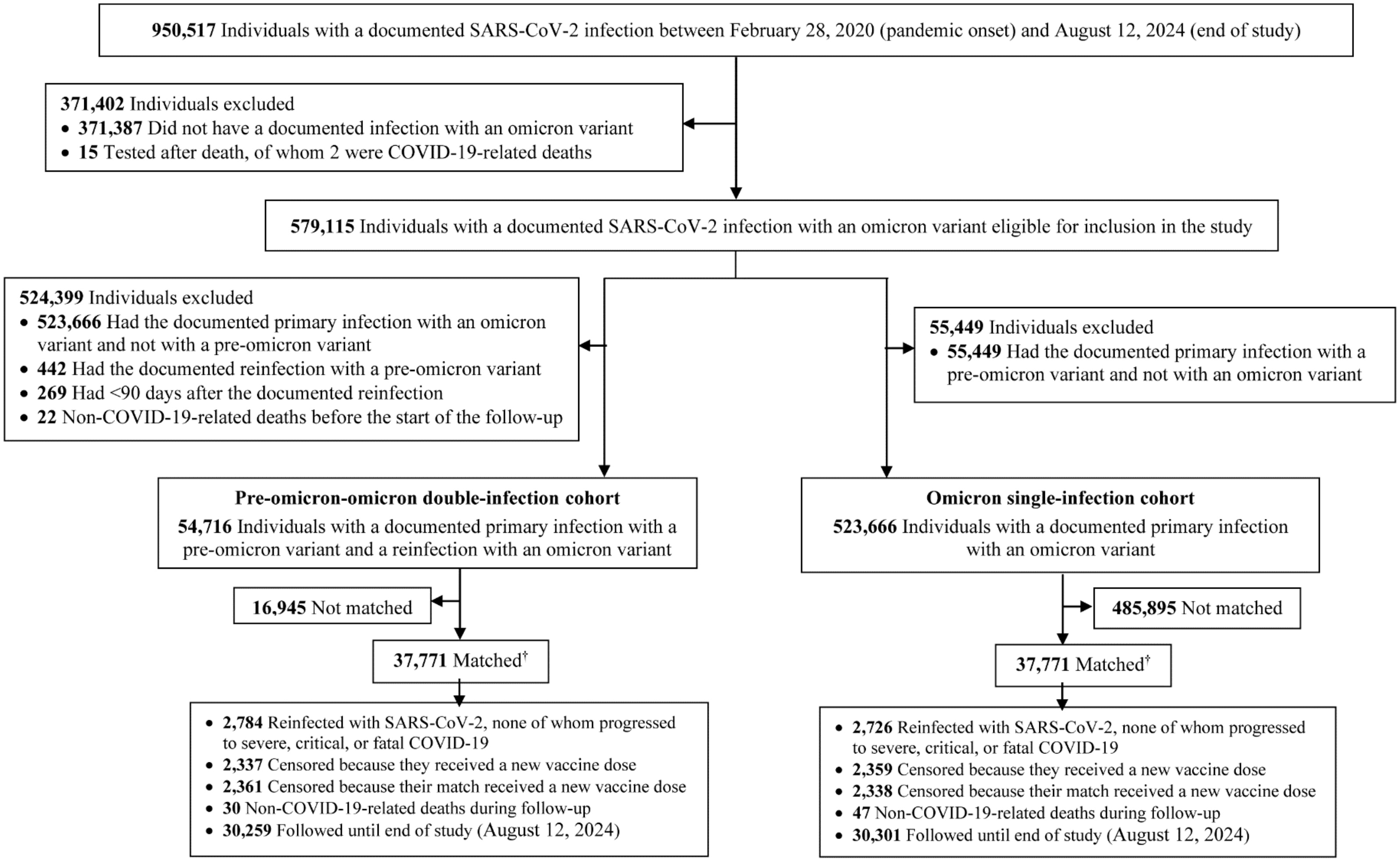

Abbreviations: COVID-19, coronavirus diseases 2019, PCR, polymerase chain reaction, SARS-CoV-2 severe acute respiratory syndrome coronavirus 2.

*A reinfection was defined as a positive SARS-CoV-2 test that is ≥90 days after the first positive test.

†Persons in the pre-omicron-omicron double-infection cohort were matched exactly one-to-one to persons in the omicron single-infection cohort by sex, 10-year age group, nationality, number of coexisting conditions, number of vaccine doses, vaccine type, in addition to testing method (PCR versus rapid-antigen testing), reason for testing, and calendar week of the SARS-CoV-2-positive test defining the reinfection with omicron for the pre-omicron-omicron double-infection cohort and defining the primary infection with omicron for the omicron single-infection cohort. Each matched pair was followed from 90 days after the date of the reinfection for the individual in the pre-omicron-omicron double-infection cohort.

Supplementary Fig. 8. Sensitivity analysis showing the adjusted cumulative incidence of SARS-CoV-2 reinfection in the studies comparing the incidence of SARS-CoV-2 reinfection between A) the omicron double-infection cohort and the omicron single-infection cohort, B) the omicron double-infection cohort and the pre-omicron-omicron double-infection cohort, and C) the pre-omicron-omicron double-infection cohort and the omicron single-infection cohort.

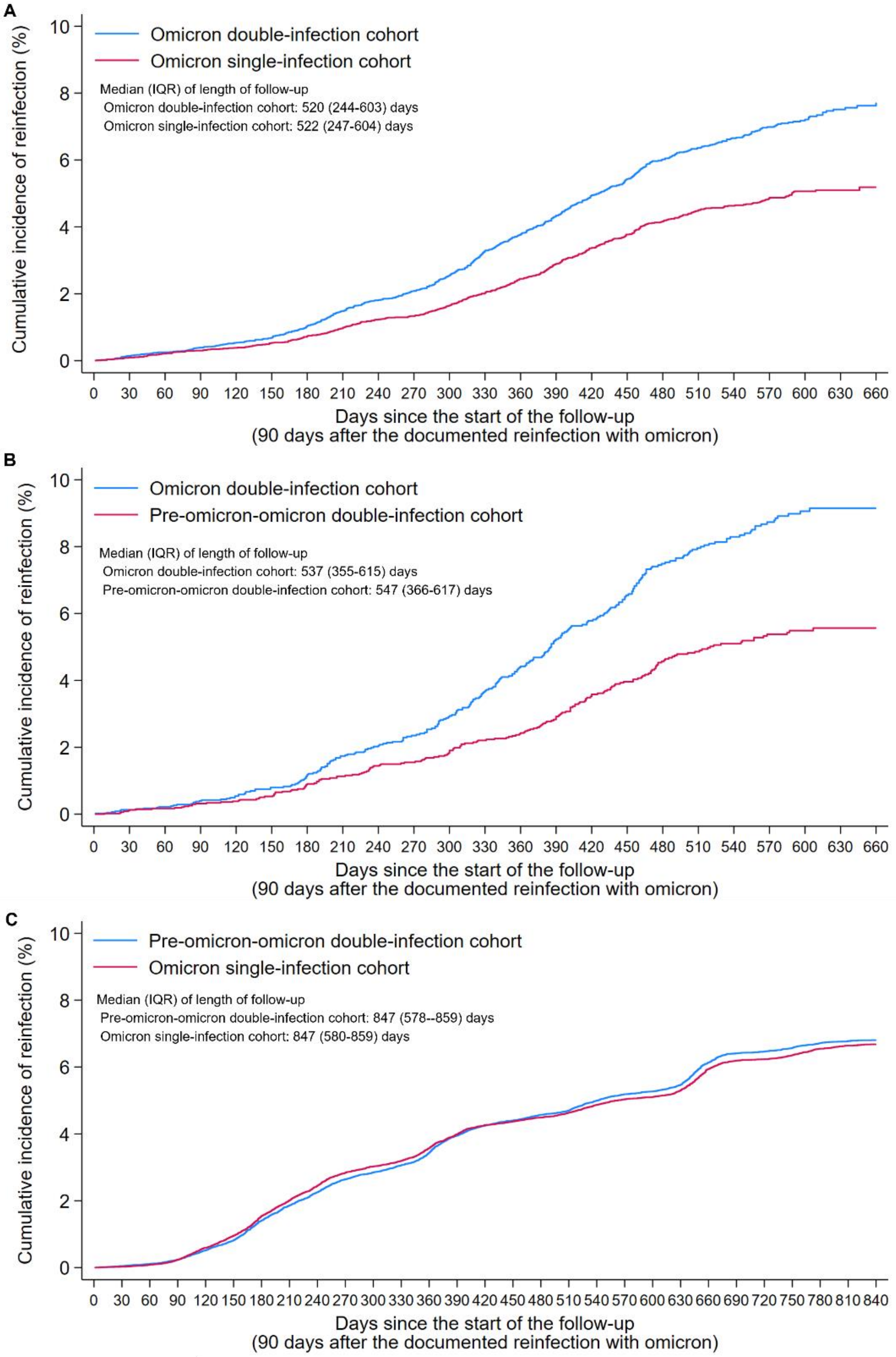

Abbreviations: IQR, interquartile range.

Supplementary Fig. 9. Sensitivity analysis of study outcomes for unvaccinated and vaccinated subgroups using interaction terms. Hazard ratios for the incidence of SARS-CoV-2 reinfection, stratified by vaccination status, in the studies comparing the incidence of SARS-CoV-2 reinfection between A) the omicron double-infection cohort and the omicron single-infection cohort, B) the omicron double-infection cohort and the pre-omicron-omicron double-infection cohort, and C) the pre-omicron-omicron double-infection cohort and the omicron single-infection cohort. Error bars indicate the corresponding 95% confidence intervals.

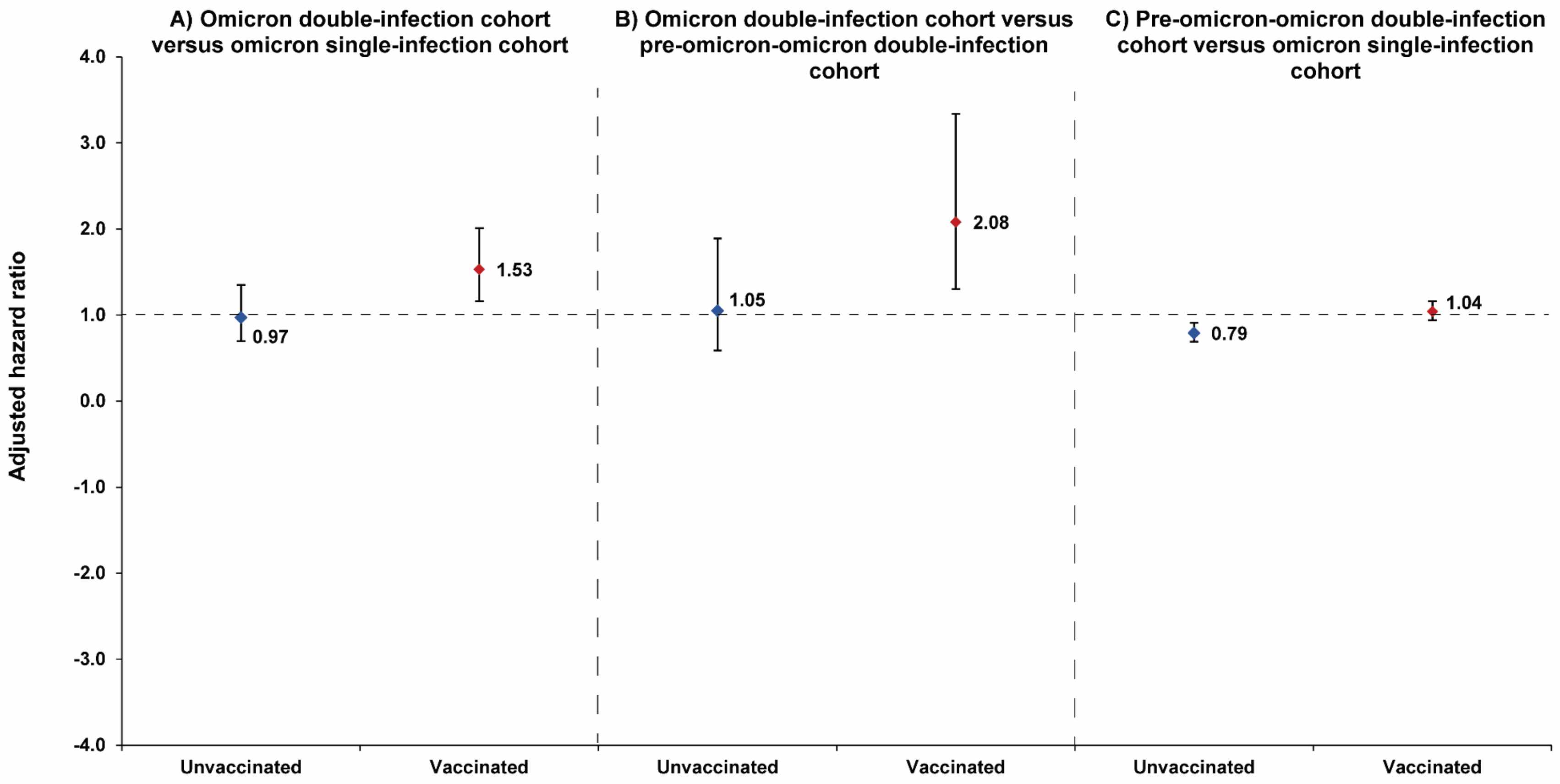

Supplementary References

1 Altarawneh, H. N. *et al.* Effects of Previous Infection and Vaccination on Symptomatic Omicron Infections. *N Engl J Med* **387**, 21-34, doi:10.1056/NEJMoa2203965 (2022).

2 Abu-Raddad, L. J. *et al.* Characterizing the Qatar advanced-phase SARS-CoV-2 epidemic. *Sci Rep* **11**, 6233, doi:10.1038/s41598-021-85428-7 (2021).

3 Chemaitelly, H. *et al.* Short- and longer-term all-cause mortality among SARS-CoV-2- infected individuals and the pull-forward phenomenon in Qatar: a national cohort study. *Int J Infect Dis* **136**, 81-90, doi:10.1016/j.ijid.2023.09.005 (2023).

4 Altarawneh, H. N. *et al.* Protection against the Omicron Variant from Previous SARS-CoV-2 Infection. *N Engl J Med* **386**, 1288-1290, doi:10.1056/NEJMc2200133 (2022).

5 Mahmoud, M. A. *et al.* SARS-CoV-2 infection and effects of age, sex, comorbidity, and vaccination among older individuals: A national cohort study. *Influenza Other Respir Viruses* **17**, e13224, doi:10.1111/irv.13224 (2023).

6 Abu-Raddad, L. J., Chemaitelly, H., Bertollini, R. & National Study Group for Covid Vaccination. Effectiveness of mRNA-1273 and BNT162b2 Vaccines in Qatar. *N Engl J Med* **386**, 799-800, doi:10.1056/NEJMc2117933 (2022).

7 Planning and Statistics Authority-State of Qatar. Qatar Monthly Statistics. Available from: https://www.psa.gov.qa/en/pages/default.aspx. Accessed on: May 26, 2020. (2020).

8 Chemaitelly, H., Bertollini, R., Abu-Raddad, L. J. & National Study Group for Covid Epidemiology. Efficacy of Natural Immunity against SARS-CoV-2 Reinfection with the Beta Variant. *N Engl J Med* **385**, 2585-2586, doi:10.1056/NEJMc2110300 (2021).

9 Chemaitelly, H. *et al.* Protection from previous natural infection compared with mRNA vaccination against SARS-CoV-2 infection and severe COVID-19 in Qatar: a retrospective cohort study. *Lancet Microbe* **3**, e944-e955, doi:10.1016/S2666-5247(22)00287-7 (2022).

10 Chemaitelly, H. *et al.* History of primary-series and booster vaccination and protection against Omicron reinfection. *Sci Adv* **9**, eadh0761, doi:10.1126/sciadv.adh0761 (2023).

11 AlNuaimi, A. A. *et al.* All-cause and COVID-19 mortality in Qatar during the COVID-19 pandemic. *BMJ Glob Health* **8**, doi:10.1136/bmjgh-2023-012291 (2023).

12 Vogels, C., Fauver, J. & Grubaugh, N. Multiplexed RT-qPCR to screen for SARS-COV-2 B.1.1.7, B.1.351, and P.1 variants of concern V.3. dx.doi.org/10.17504/protocols.io.br9vm966. (2021).

13 Abu-Raddad, L. J., Chemaitelly, H., Butt, A. A. & National Study Group for Covid Vaccination. Effectiveness of the BNT162b2 Covid-19 Vaccine against the B.1.1.7 and B.1.351 Variants. *N Engl J Med* **385**, 187-189, doi:10.1056/NEJMc2104974 (2021).

14 Chemaitelly, H. *et al.* mRNA-1273 COVID-19 vaccine effectiveness against the B.1.1.7 and B.1.351 variants and severe COVID-19 disease in Qatar. *Nat Med* **27**, 1614-1621, doi:10.1038/s41591-021-01446-y (2021).

15 National Project of Surveillance for Variants of Concern and Viral Genome Sequencing. *Qatar viral genome sequencing data. Data on randomly collected samples. https://www.gisaid.org/phylodynamics/global/nextstrain/*, <https://www.gisaid.org/phylodynamics/global/nextstrain/> (2021).

16 Benslimane, F. M. *et al.* One Year of SARS-CoV-2: Genomic Characterization of COVID-19 Outbreak in Qatar. *Front Cell Infect Microbiol* **11**, 768883, doi:10.3389/fcimb.2021.768883 (2021).

17 Hasan, M. R. *et al.* Real-Time SARS-CoV-2 Genotyping by High-Throughput Multiplex PCR Reveals the Epidemiology of the Variants of Concern in Qatar. *Int J Infect Dis* **112**, 52-54, doi:10.1016/j.ijid.2021.09.006 (2021).

18 Saththasivam, J. *et al.* COVID-19 (SARS-CoV-2) outbreak monitoring using wastewater-based epidemiology in Qatar. *Sci Total Environ* **774**, 145608, doi:10.1016/j.scitotenv.2021.145608 (2021).
19 El-Malah, S. S. *et al.* Application of human RNase P normalization for the realistic estimation of SARS-CoV-2 viral load in wastewater: A perspective from Qatar wastewater surveillance. *Environ Technol Innov* **27**, 102775, doi:10.1016/j.eti.2022.102775 (2022).
20 El-Malah, S. S. *et al.* Leveraging wastewater surveillance for managing the spread of SARS-CoV-2 and concerned pathogens during FIFA World Cup Qatar 2022. *Heliyon* **10**, e30267, doi:10.1016/j.heliyon.2024.e30267 (2024).
21 Tang, P. *et al.* BNT162b2 and mRNA-1273 COVID-19 vaccine effectiveness against the SARS-CoV-2 Delta variant in Qatar. *Nat Med* **27**, 2136-2143, doi:10.1038/s41591-021-01583-4 (2021).
22 Chemaitelly, H. *et al.* Duration of mRNA vaccine protection against SARS-CoV-2 Omicron BA.1 and BA.2 subvariants in Qatar. *Nat Commun* **13**, 3082, doi:10.1038/s41467-022-30895-3 (2022).
23 Qassim, S. H. *et al.* Effects of BA.1/BA.2 subvariant, vaccination and prior infection on infectiousness of SARS-CoV-2 omicron infections. *J Travel Med* **29**, doi:10.1093/jtm/taac068 (2022).
24 Altarawneh, H. N. *et al.* Protective Effect of Previous SARS-CoV-2 Infection against Omicron BA.4 and BA.5 Subvariants. *N Engl J Med* **387**, 1620-1622, doi:10.1056/NEJMc2209306 (2022).
25 Chemaitelly, H. *et al.* Protection against Reinfection with the Omicron BA.2.75 Subvariant. *N Engl J Med* **388**, 665-667, doi:10.1056/NEJMc2214114 (2023).
26 Chemaitelly, H. *et al.* Protection of natural infection against reinfection with SARS-CoV-2 JN.1 variant. *medRxiv*, 2024.2002.2022.24303193, doi:10.1101/2024.02.22.24303193 (2024).
27 World Health Organization (WHO). Living guidance for clinical management of COVID-19. Aavailable from: https://www.who.int/publications/i/item/WHO-2019-nCoV-clinical-2021-2. Accessed on: February 27, 2023. (2021).
28 World Health Organization (WHO). International Guidelines for Certification and Classification (Coding) of COVID-19 as Cause of Death. Available from: https://www.who.int/publications/m/item/international-guidelines-for-certification-and-classification-(coding)-of-covid-19-as-cause-of-death. Accessed on: February 27, 2023. (2020).
29 Chemaitelly, H. *et al.* Turning point in COVID-19 severity and fatality during the pandemic: a national cohort study in Qatar. *BMJ Public Health* **1**, e000479, doi:10.1136/bmjph-2023-000479 (2023).
30 Abu-Raddad, L. J. *et al.* Severity, Criticality, and Fatality of the Severe Acute Respiratory Syndrome Coronavirus 2 (SARS-CoV-2) Beta Variant. *Clin Infect Dis* **75**, e1188-e1191, doi:10.1093/cid/ciab909 (2022).
31 Abu-Raddad, L. J., Chemaitelly, H., Bertollini, R. & National Study Group for Covid Epidemiology. Severity of SARS-CoV-2 Reinfections as Compared with Primary Infections. *N Engl J Med* **385**, 2487-2489, doi:10.1056/NEJMc2108120 (2021).
32 Al-Thani, M. H. *et al.* SARS-CoV-2 Infection Is at Herd Immunity in the Majority Segment of the Population of Qatar. *Open Forum Infect Dis* **8**, ofab221, doi:10.1093/ofid/ofab221 (2021).
33 Ayoub, H. H. *et al.* Mathematical modeling of the SARS-CoV-2 epidemic in Qatar and its impact on the national response to COVID-19. *J Glob Health* **11**, 05005, doi:10.7189/jogh.11.05005 (2021).
34 Coyle, P. V. *et al.* SARS-CoV-2 seroprevalence in the urban population of Qatar: An analysis of antibody testing on a sample of 112,941 individuals. *iScience* **24**, 102646, doi:10.1016/j.isci.2021.102646 (2021).

35 Jeremijenko, A. *et al.* Herd Immunity against Severe Acute Respiratory Syndrome Coronavirus 2 Infection in 10 Communities, Qatar. *Emerg Infect Dis* **27**, 1343-1352, doi:10.3201/eid2705.204365 (2021).
36 Abu-Raddad, L. J. *et al.* Pfizer-BioNTech mRNA BNT162b2 Covid-19 vaccine protection against variants of concern after one versus two doses. *J Travel Med* **28**, doi:10.1093/jtm/taab083 (2021).
37 Chemaitelly, H. *et al.* Waning of BNT162b2 Vaccine Protection against SARS-CoV-2 Infection in Qatar. *N Engl J Med* **385**, e83, doi:10.1056/NEJMoa2114114 (2021).
38 Abu-Raddad, L. J. *et al.* Effect of mRNA Vaccine Boosters against SARS-CoV-2 Omicron Infection in Qatar. *N Engl J Med* **386**, 1804-1816, doi:10.1056/NEJMoa2200797 (2022).
39 Chemaitelly, H. *et al.* Long-term COVID-19 booster effectiveness by infection history and clinical vulnerability and immune imprinting: a retrospective population-based cohort study. *Lancet Infect Dis* **23**, 816-827, doi:10.1016/S1473-3099(23)00058-0 (2023).
40 Chemaitelly, H. *et al.* Duration of immune protection of SARS-CoV-2 natural infection against reinfection. *J Travel Med* **29**, doi:10.1093/jtm/taac109 (2022).
