# Supplementary material for: Immune histories and natural infection protection during the omicron era
Source: Commun Med (Lond). 2025 Jul 1;5:262. doi: 10.1038/s43856-025-00974-9 (PMC12215862; doi:10.1038/s43856-025-00974-9)
Supplement: Supplementary file 3 — Description of Additional Supplementary files [file 43856_2025_974_MOESM3_ESM.pdf]

Description of Additional Supplementary files

File name: Supplementary Data 1

Description: Baseline characteristics of matched cohorts used to compare SARS-CoV-2 reinfection rates across cohorts with different infection histories.

File name: Supplementary Data 2

Description: Cumulative incidence of SARS-CoV-2 reinfection in the studies comparing the incidence of SARS-CoV-2 reinfection across cohorts with different infection histories.

File name: Supplementary Data 3

Description: Hazard ratios for the incidence of SARS-CoV-2 reinfection, stratified by vaccination status, based on matched cohorts with different infection histories.
