# Supplementary material for: Immune histories and natural infection protection during the omicron era
Source: Commun Med (Lond). 2025 Jul 1;5:262. doi: 10.1038/s43856-025-00974-9 (PMC12215862; doi:10.1038/s43856-025-00974-9)
Supplement: Supplementary file 7 — Reporting summary [file 43856_2025_974_MOESM7_ESM.pdf]

nature portfolio

Corresponding author(s): Laith J. Abu-Raddad

Last updated by author(s): Jun 6, 2025

Reporting Summary

Nature Portfolio wishes to improve the reproducibility of the work that we publish. This form provides structure for consistency and transparency in reporting. For further information on Nature Portfolio policies, see our Editorial Policies and the Editorial Policy Checklist.

Statistics

For all statistical analyses, confirm that the following items are present in the figure legend, table legend, main text, or Methods section.

n/a	Confirmed	
☐	☒	The exact sample size (*n*) for each experimental group/condition, given as a discrete number and unit of measurement
☐	☒	A statement on whether measurements were taken from distinct samples or whether the same sample was measured repeatedly
☒	☐	The statistical test(s) used AND whether they are one- or two-sided<br>*Only common tests should be described solely by name; describe more complex techniques in the Methods section.*
☐	☒	A description of all covariates tested
☐	☒	A description of any assumptions or corrections, such as tests of normality and adjustment for multiple comparisons
☐	☒	A full description of the statistical parameters including central tendency (e.g. means) or other basic estimates (e.g. regression coefficient) AND variation (e.g. standard deviation) or associated estimates of uncertainty (e.g. confidence intervals)
☒	☐	For null hypothesis testing, the test statistic (e.g. *F*, *t*, *r*) with confidence intervals, effect sizes, degrees of freedom and *P* value noted<br>*Give P values as exact values whenever suitable.*
☒	☐	For Bayesian analysis, information on the choice of priors and Markov chain Monte Carlo settings
☒	☐	For hierarchical and complex designs, identification of the appropriate level for tests and full reporting of outcomes
☐	☒	Estimates of effect sizes (e.g. Cohen's *d*, Pearson's *r*), indicating how they were calculated

Our web collection on statistics for biologists contains articles on many of the points above.

Software and code

Policy information about availability of computer code

Data collection	Data were available to authors through .csv files downloaded from the CERNER database system.
Data analysis	Standard epidemiological analyses were conducted using standard commands in STATA/SE 18.0. These commands have been published at: https://github.com/IDEGWCMQ/Cohort/blob/main/Cohort-Code.do

For manuscripts utilizing custom algorithms or software that are central to the research but not yet described in published literature, software must be made available to editors and reviewers. We strongly encourage code deposition in a community repository (e.g. GitHub). See the Nature Portfolio guidelines for submitting code & software for further information.

Data

Policy information about availability of data

All manuscripts must include a data availability statement. This statement should provide the following information, where applicable:

- Accession codes, unique identifiers, or web links for publicly available datasets
- A description of any restrictions on data availability
- For clinical datasets or third party data, please ensure that the statement adheres to our policy

The dataset of this study is a property of the Qatar Ministry of Public Health that was provided to the researchers through a restricted-access agreement that prevents sharing the dataset with a third party or publicly. The data are available under restricted access for preservation of confidentiality of patient data. Access can be obtained through a direct application for data access to Her Excellency the Minister of Public Health (https://emsfsa.moph.gov.qa/en/Pages/eservices.aspx).

The raw data are protected and are not available due to data privacy laws. Data were available to authors through .csv files where information has been downloaded from the CERNER database system (no links/accession codes were available to authors). Aggregate data are available within the manuscript and its Supplementary information.

Research involving human participants, their data, or biological material

Policy information about studies with human participants or human data. See also policy information about sex, gender (identity/presentation), and sexual orientation and race, ethnicity and racism.

Reporting on sex and gender	The study populations are balanced by sex (please see Supplementary Data 1). Sex is as recorded in the integrated nationwide digital-health information platform, which is based on the Qatar Identity Card.
Reporting on race, ethnicity, or other socially relevant groupings	The study populations are balanced across nationality groups. Nationality, age, and sex provide a powerful proxy for occupation and socio-economic status in Qatar as evidenced by earlier studies in this population.
Population characteristics	The demographic characteristics of the study populations can be found in Supplementary Data 1.
Recruitment	Three national, matched, retrospective cohort studies were conducted in Qatar from February 28, 2020, to August 12, 2024 to examine how different natural infection histories, in addition to an omicron infection, may influence protection against subsequent omicron reinfection. The first study compared omicron reinfection rates between individuals with two omicron infections (omicron double-infection cohort) and those with one (omicron single-infection cohort). The second study compared the omicron double-infection cohort with individuals who had a pre-omicron infection followed by an omicron reinfection (Pre-omicron-omicron double-infection cohort). The third study compared the pre-omicron-omicron double-infection cohort with the omicron single-infection cohort. COVID-19 laboratory testing, clinical infection data, severity, hospitalization, vaccination, and related demographic details were extracted from the integrated nationwide digital-health information platform that hosts the national, federated SARS-CoV-2 databases. These databases are complete with no missing information for PCR testing, medically-supervised rapid antigen testing, COVID-19 vaccinations, COVID-19 hospitalizations and deaths, and basic demographic details, and have captured all SARS-CoV-2-related data since epidemic onset. This study was conducted on the entire resident population of Qatar. Any individual who had an omicron infection was eligible for inclusion in the study. SARS-CoV-2 testing was extensive in Qatar until October 31, 2022, with nearly 5% of the population being tested every week, primarily for routine purposes such as screening or meeting travel-related requirements. Subsequently, testing rates decreased, with less than 1% of the population being tested per week. The majority of infections during the pandemic were diagnosed through routine testing rather than symptomatic presentation.
Ethics oversight	The study was approved by the Hamad Medical Corporation and Weill Cornell Medicine-Qatar Institutional Review Boards with waiver of informed consent.

Note that full information on the approval of the study protocol must also be provided in the manuscript.

Field-specific reporting

Please select the one below that is the best fit for your research. If you are not sure, read the appropriate sections before making your selection.

☒ Life sciences ☐ Behavioural & social sciences ☐ Ecological, evolutionary & environmental sciences

For a reference copy of the document with all sections, see nature.com/documents/nr-reporting-summary-flat.pdf

Life sciences study design

All studies must disclose on these points even when the disclosure is negative.

Sample size	COVID-19 laboratory testing, clinical infection data, severity, hospitalization, vaccination, and related demographic details were extracted from the integrated nationwide digital-health information platform that hosts the national, federated SARS-CoV-2 databases. These databases are complete and have captured SARS-CoV-2-related data since epidemic onset. The data is for the entire national population and includes every individual tested for SARS-CoV-2 in any facility in Qatar. The sample size varied depending on individuals who met the eligibility criteria for each cohort. Individuals in the omicron double-infection cohort needed to have a primary omicron infection followed by an omicron reinfection. Individuals in the pre-omicron-omicron double-infection cohort needed to have a primary pre-omicron infection followed by an omicron reinfection. Individuals in the omicron single-infection cohort needed to have a primary omicron infection. Cohorts were matched exactly one-to-one by sex, 10-year age group, nationality, number of coexisting conditions, number of vaccine doses, and vaccine type, as ascertained at the start of follow-up. Matching was also performed based on the testing method (polymerase chain reaction versus rapid-antigen testing), reason for testing, and calendar week of the SARS-CoV-2 test that defined reinfection for the omicron double-infection cohort, reinfection for the pre-omicron-omicron double-infection cohort, and primary infection for the omicron single-infection cohort. Given that the sample sizes were based on the entire national population with only individuals that do not fit the eligibility criteria excluded, the sample size for each study can be considered sufficient. Detailed sample sizes can be found in Supplementary Figs. 2, 6 and 7 and Supplementary Data 1.
Data exclusions	Exclusion criteria were specified a priori. Cohorts were formed based on existence of a primary omicron infection followed by an omicron reinfection for the omicron double-infection cohort, a primary pre-omicron infection followed by an omicron reinfection for the pre-omicron-omicron double-infection cohort, and a primary infection with omicron for the omicron single-infection cohort. Following the conventional definition, a reinfection was defined as a documented infection occurring ≥90 days after a previous infection to avoid misclassification of prolonged test positivity as reinfection. Cohorts were matched exactly one-to-one by sex, 10-year age group, nationality, number of coexisting

conditions, number of vaccine doses, and vaccine type, as ascertained at the start of follow-up. Matching was also performed based on the testing method (polymerase chain reaction versus rapid-antigen testing), reason for testing, and calendar week of the SARS-CoV-2 test that defined reinfection for the omicron double-infection cohort, reinfection for the pre-omicron-omicron double-infection cohort, and primary infection for the omicron single-infection cohort. Iterative matching was implemented to ensure that, at the start of follow-up, individuals were alive and had the same vaccine type and number of doses as their match.

Replication	To confirm the adjusted hazard ratio estimates among unvaccinated and vaccinated individuals, these outcomes were also generated using interaction terms between study cohorts and vaccination status. Cox interaction models were applied to the full cohorts to evaluate these interactions. The estimates obtained aligned with those of the main analysis.
Randomization	Not applicable as these are observational cohort studies where individuals are aware of their infection and vaccination statuses. However, individuals were selected from the entire national population, and exact matching on multiple factors was employed to ensure rigorous pairing of individuals in all studies.
Blinding	Not applicable as these are observational cohort studies where individuals are aware of their infection and vaccination statuses.

Reporting for specific materials, systems and methods

We require information from authors about some types of materials, experimental systems and methods used in many studies. Here, indicate whether each material, system or method listed is relevant to your study. If you are not sure if a list item applies to your research, read the appropriate section before selecting a response.

Materials & experimental systems

n/a	Involved in the study
☒	☐ Antibodies
☒	☐ Eukaryotic cell lines
☒	☐ Palaeontology and archaeology
☒	☐ Animals and other organisms
☒	☐ Clinical data
☒	☐ Dual use research of concern
☒	☐ Plants

Methods

n/a	Involved in the study
☒	☐ ChIP-seq
☒	☐ Flow cytometry
☒	☐ MRI-based neuroimaging
